# Single-cell landscape of immune cells during the progression from HBV infection to HBV cirrhosis and HBV-associated hepatocellular carcinoma

**DOI:** 10.3389/fimmu.2023.1320414

**Published:** 2023-12-05

**Authors:** Qingquan Bai, Runyang Li, Xiao He, Xiaoting Hong, Ying Yan, Zhengyang Zhao, Han Lin, Frank Tacke, Cornelius Engelmann, Tianhui Hu

**Affiliations:** ^1^ Department of Hepatology & Gastroenterology, Charité Universitätsmedizin Berlin, Berlin, Germany; ^2^ Cancer Research Center, School of Medicine, Xiamen University, Xiamen, China; ^3^ Department of Neurology, The First Affiliated Hospital of Shandong First Medical University & Shandong Provincial Qianfoshan Hospital, Jinan, China; ^4^ Department of Hepatic Surgery, The First Affiliated Hospital of Harbin Medical University, Harbin, China; ^5^ Berlin Institute of Health - Charité - Universitätsmedizin Berlin, Berlin, Germany; ^6^ Institute for Liver and Digestive Health, University College London, London, United Kingdom; ^7^ Shenzhen Research Institute of Xiamen University, Shenzhen, China

**Keywords:** hepatitis B, cirrhosis, HBV infection, hepatocellular carcinoma, scRNA-seq

## Abstract

**Introduction:**

Immune cells play crucial roles in the development of chronic hepatitis B virus (HBV) infection, leading to cirrhosis and hepatocellular carcinoma (HCC). However, their functions at different disease stages are not fully understood.

**Methods:**

In this study, we used single-cell RNA sequencing (scRNA-seq) to characterize the human liver immune microenvironment at different disease stages. We analyzed scRNA-seq data from 118,455 immune cells obtained from livers of six healthy individuals, four patients with HBV infection, five patients with HBV cirrhosis, and three patients with HBV-associated HCC.

**Results:**

Our results showed an accumulation of scar-associated macrophages during disease progression, and we identified two relevant immune subsets, Macrophage-CD9/IL18 and macrophage-CD9/IFI6. Macrophage-CD9/IL18 expanded from HBV infection to cirrhosis, while macrophage-CD9/IFI6 expanded from cirrhosis to HCC. We verified the existence of Macrophage-CD9/IFI6 using multiplex immunofluorescence staining. We also found an increase in cytotoxic NK Cell-GNLY during progression from cirrhosis to HCC. Additionally, the proportion of CD4 T cell-TNFAIP3, CD8 T cell-TNF (effector CD8 T cells), and CD8 T cell-CD53 increased, while the proportion of Treg cells decreased from HBV infection to cirrhosis. The proportion of Treg and CD8 T cell-LAG3 (Exhausted CD8 T cell) enhanced, while the proportion of CD8 T cell-TNF (effector CD8 T cells) decreased from cirrhosis to HCC. Furthermore, GSEA enrichment analyses revealed that MAPK, ERBB, and P53 signaling pathways in myeloid cells were gradually inhibited from HBV infection to cirrhosis and HCC.

**Discussion:**

Our study provides important insights into changes in the hepatic immune environment during the progression of HBV-related liver disease, which may help improve the management of HBV-infected liver diseases.

## Introduction

Hepatitis B virus (HBV) is a communicable chronic disease that poses a significant threat to public health. It is estimated that approximately 296 million people worldwide are infected with HBV, resulting in 1 million deaths annually (1). HBV is a hepatotropic virus that causes hepatocyte injury, immune cell infiltration, and liver fibrosis (2). As a result, patients may develop liver cirrhosis and hepatocellular carcinoma (HCC) (3). In HBV-infected individuals, approximately 90% of HCC cases develop on a background of cirrhosis, and 4% of patients with cirrhosis progress to HCC each year (4, 5).

Recent advances in high-throughput RNA sequencing at the single-cell level have enabled the identification of global patterns of stochastic gene expression. Single-cell RNA sequencing (scRNA-seq) is particularly well-suited to resolving immune cellular complexity and heterogeneity, identifying new cell subsets, and delineating underlying cell lineage relationships (6). Although scRNA-seq studies have been performed on liver tissue from patients with acute HBV infection (7), non-hepatitis cirrhosis (8), and HCC (9, 10), a comparison across disease stages in a single study is still lacking. Therefore, in this study, we used scRNA-seq to construct an unbiased and comprehensive intrahepatic immune cell atlas and compared the changes in immune cells from healthy liver to HBV infection, HBV cirrhosis, and HBV HCC.

## Materials and methods

### Study subjects

Our study was approved by the First Affiliated Hospital of Harbin Medical University Ethics Review Center (approval number. 2021179). Informed consents were obtained from all patients and donors. Liver tissue samples from healthy donors (n = 2) and patients with HBV cirrhosis (n = 2) were collected during orthotopic liver transplantation at the Department of Liver Surgery. Healthy samples are small samples for testing organ quality before liver transplantation of healthy organs. Informed consent was obtained from all the patients and donors. Before interrupting hepatic vascular inflow, wedge biopsies of non-ischemic fresh liver tissue (2~3 grams per liver) were collected from all livers during the operation. We obtained single-cell transcriptome data of 45,993 immune cells from these four samples. With the permission and help from China National Center for Bioinformation (CNCB), we also obtained single-cell transcript transcriptome of 4 healthy livers and 4 HBV infection livers from HRA001730, and 3 HBV cirrhotic livers and 3 HBV HCC samples from HRA000069. In total, we analyzed 118,455 immune cells from healthy livers (n = 6), HBV infection livers (n = 4), HBV cirrhotic livers (n = 5), and HBV HCC samples (n = 3). Tissue for multiplex immunofluorescence was also obtained from the First Affiliated Hospital of Harbin Medical University. The clinical characteristics of these patients are summarized in **Supplementary Table 1**.

### ScRNA-seq based on droplet generation

Cells were processed through the 10X Genomics Single Cell Platform by using the Next GEM Sing cell v3.1 library kit (10X Genomics, PN-1000157, US), Next GEM Gel Beads v3.1 (10X Genomics, PN-2000164, US) and Next GEM chip G (10X Genomics, PN-2000177, US). The number of sample cells on a 10x chip was 16,000. The gel beads and single cells with barcodes and primers were wrapped in oil droplets in the Chromium system to form the oil-in-water structure of a single cell. The cells were lysed in the water in an oil structure to obtain the mRNA, which was subsequently retro transcribed to cDNA. The library was sequenced and detected combined with the MGISEQ-2000 sequencing platform.

### Cell clustering

Cell clustering was performed using UMAP (Uniform Manifold Approximation and Projection for Dimension Reduction) for non-linear dimension reduction and density-based clustering. Each point in the plot represented a single cell, and the closed spatial distance between cells indicated similar gene expression patterns. Colors were determined by the classification result of a graph-based clustering algorithm, which considers that the cells in the same cluster have the closest expression pattern. To conserve computational resources, we reduced the number of normal samples in T cell clustering analysis.

### Multiplex immunofluorescence

The multiplex immunofluorescence staining method (11) allows to analyze the differential expression of protein markers with multiple markers in the same section. Briefly, the slides with liver section were baked at a constant temperature of 63°C in an oven (Haiyiheng Scientific Instruments, PH-070A, China) for 2 h. After deparaffinization with xylene and hydration with a gradual series of ethanol, antigen retrieval was performed with a ready-to-use antigen retrieval solution in a microwave for 3 min at high voltage and subsequently for another 15 min at low voltage. Subsequently, sections were blocked with goat serum (Bosterbio, AR1009, China) for 30 min. After washing with TBST, the sections were incubated with primary antibodies (CD9, Proteintech, 60232-1-Ig, China; IL-18, Proteintech, 10663-1-AP, China) for 1 h at room temperature, followed by incubation with corresponding secondary antibodies for 10 min at room temperature. Cy3 (Bosterbio, BA1032, China) and FITC (Bosterbio, BA1101, China) were used to visualize the markers. The sections were counterstained with DAPI (Thermo Scientific, 62248, USA) for 10 min at room temperature and mounted with the VECTASHIELD hardest antifade mounting medium (Vector Labs, H-1400, USA). The entire scans were visualized with a Tissue CaseViewer (3DHISTECH, Hungary), and five different images were randomly selected from each sample for analysis.

## Result

### Single-cell atlas of immune cells from HBV infection to HBV cirrhosis and HBV HCC

**Figure 1:**
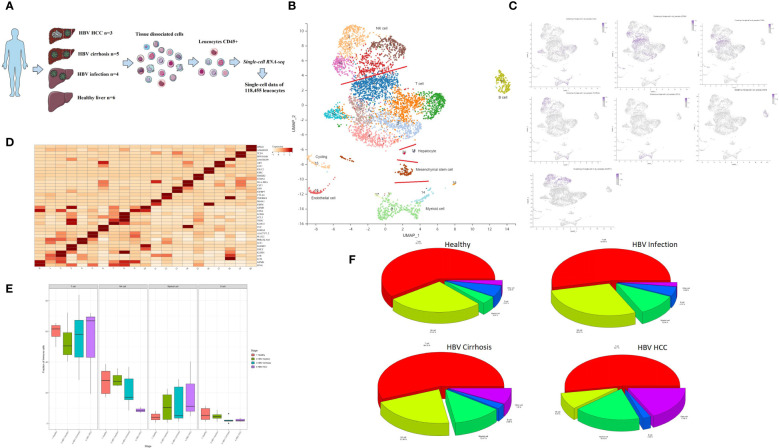
Single-cell atlas of healthy, HBV-infected, HBV-cirrhotic, and HBV-HCC livers. **(A)** Workflow of isolation, sorting, and sc-RNASeq of CD45+ leukocytes from healthy liver to HBV infection, HBV cirrhosis, and HBV HCC using flow cytometry and 10× Genomics platform. **(B)** Clustering analysis of all the CD45+ leukocytes. **(C)** The expression of CD4, CD8A, CD8B, CD14, CD19, FCGR3A (CD16), and KLRF1 in UMAP plot. **(D)** The two most significant marker genes in each cluster displayed in the dot plot. **(E)** The proportion of T cells, NK cells, myeloid cells, and B cells from healthy liver to HBV infection, HBV cirrhosis, and HBV HCC. **(F)** Pie chart of the average proportion of T cells, NK cells, myeloid cells, and B cells from healthy liver to HBV infection, HBV cirrhosis, and HBV HCC.

Hepatic CD45+ leukocytes were isolated by fluorescence-activated cell sorting (FACS) prior to single-cell RNA sequencing (scRNA-seq) analysis (**Figure 1A**). A total of 45,993 immune cells were analyzed, and with the aid of public data (7, 10), we ultimately analyzed 118,455 immune cells from healthy livers (n = 6), HBV infection livers (n = 4), HBV cirrhotic livers (n = 5), and HBV HCC samples (n = 3).

Principal component analysis (PCA) was performed, followed by dimensionality reduction using the UMAP algorithm with 15 principal components. Based on the most significant marker genes (**Figure 1B**), twenty cell clusters were identified and classified into four major cell subtypes: NK cells, T cells, B cells, and myeloid cells. The characteristic genes of the major cell clusters were CD4/CD8A/CD8B for T cells, CD14/FCGR3A(CD16) for myeloid cells, CD19 for B cells, and KLRF1 for NK cells (**Figure 1C**). The two most significant marker genes are shown in **Figure 1D**. The proportion of T cells, NK cells, myeloid cells, and B cells is shown in **Figures 1E, F**. Contamination with non-hematopoietic cells, such as cycling cells, endothelial cells, mesenchymal stem cells, and hepatocytes, which were identified by SCSA software (12), was low, with an overall proportion of 5.1% of the total number of cells.

### Macrophage-CD9/IL18 expanded from HBV infection to cirrhosis and macrophage-CD9/IFI6 expanded from cirrhosis to HCC

**Figure 2:**
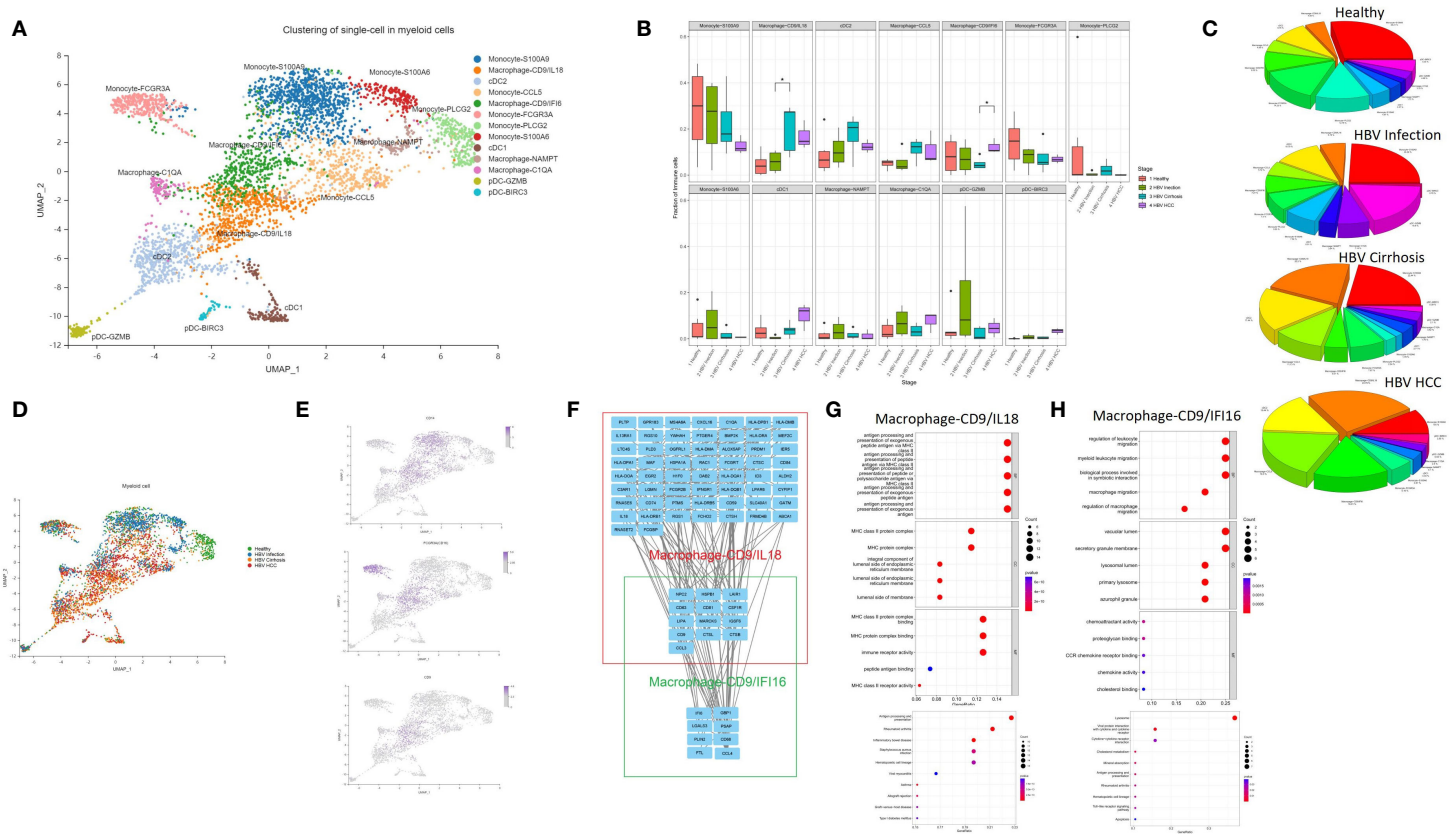
The subtypes of myeloid cells. **(A)** Clustering analysis of all the myeloid cells. **(B)** The proportion of myeloid cells from healthy liver to HBV infection, HBV cirrhosis, and HBV HCC located in different clusters. *p<0.05. **(C)** Pie chart of the average proportion of each subtype from healthy liver to HBV infection, HBV cirrhosis, and HBV HCC. **(D)** The expression of CD14, FCGR3A (CD16), and CD9 in UMAP plot. **(E)** Clustering analysis of the myeloid cells from healthy liver to HBV infection, HBV cirrhosis, and HBV HCC. **(F)** PPI network of the marker genes of Macrophage-CD9/IFI6 and Macrophage-CD9/IL18. **(G)** GO and KEGG enrichment analysis of Macrophage-CD9/IL18 marker genes. **(H)** KEGG and KEGG enrichment analysis of Macrophage-CD9/IFI6 marker genes.

Myeloid cells contribute to the development and maintenance of liver disease (13). In this study, we conducted an in-depth analysis of myeloid subclusters and identified thirteen subclusters, including macrophages, monocytes, plasmacytoid dendritic cells (pDCs), and conventional dendritic cells (cDCs) (**Figure 2A**). We calculated the proportion of each cluster in different HBV disease stages (**Figures 2B–D**). Monocytes comprised of five subclusters that were labeled according to characteristic marker gene expression: Monocyte-S100A9, Monocyte-PLCG2, Monocyte-CCL5, Monocyte-S100A6, and Monocyte-FCGR3A. Both Monocyte-S100A9 and Monocyte-S100A6 were considered CD14 classical monocyte (**Figure 2E**), enriched in the expression of S100A4, S100A6, S100A9, and S100A11. Monocyte-FCGR3A was considered a non-classical CD16 monocyte showing high expression of FCGR3A (CD16) (**Figure 2E**).

Macrophages were divided into four subclusters, Macrophage-CD9/IL18, Macrophage-CD9/IFI6, Macrophage-NAMPT, and Macrophage-C1QA. Macrophage-CD9/IL18 and Macrophage-CD9/IFI6 have the same marker gene, CD9 (**Figure 2B**). Previous studies identified a macrophage subcluster enriched by CD9 that inhabits the fibrotic niche in liver cirrhosis, termed as scar-associated macrophages (8). Both Macrophage-CD9/IL18 and Macrophage-CD9/IFI6 share characteristics of scar-associated macrophages. Macrophage-CD9/IL18 expanded from HBV infection to HBV cirrhosis (P<0.05) (**Figure 2B**), while Macrophage-CD9/IFI6 were increasingly recognized during HCC development (P<0.05) (**Figure 2B**).

To further study the phenotype of Macrophage-CD9/IL18 and Macrophage-CD9/IFI6, we constructed a PPI network (**Figure 2F**) and performed the Kyoto Encyclopedia of Genes and Genomes (KEGG) pathway and Gene Ontology (GO) enrichment analysis of the marker genes of Macrophage-CD9/IL18 and Macrophage-CD9/IFI6 (**Figures 2G, H**). Macrophage-CD9/IL18 were characterized by MHC class II proteins, viral protein interaction, and antigen processing and presentation in the enrichment analysis (**Figure 2G**). Macrophage-CD9/IFI6 were more related to leukocyte migration, viral protein interaction with cytokines and cytokine receptors, and lysosomes (**Figures 2H**).

### Different cell communication roles of macrophage-CD9/IFI6 and -CD9/IL18 during HBV infection to cirrhosis and HCC

**Figure 3:**
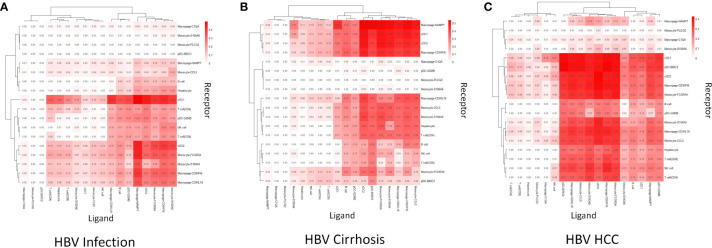
Cell communication analysis. **(A)** The strength of cell communication of immune cells in HBV infection. **(B)** The strength of cell communication of immune cells in HBV cirrhosis. **(C)** The strength of cell communication of immune cells in HBV HCC.

To reveal immune cell communication during the progression from HBV infection to HBV HCC, receptor-ligand interactions were analyzed through Cellchat. Macrophage-CD9/IL18 and Macrophage-CD9/IFI6 were predicted to interact strongly with cDC1 in HBV infection (**Figure 3A**). However, cell-cell interactions changed in different disease stages so that both scar-associated macrophage subclusters were closely linked with pDC-BIRC3 in HBV cirrhosis (**Figure 3B**), and with cDC1 and cDC2 in HBV HCC (**Figure 3C**).

### Macrophage-CD9/IL18 in different liver diseases

**Figure 4:**
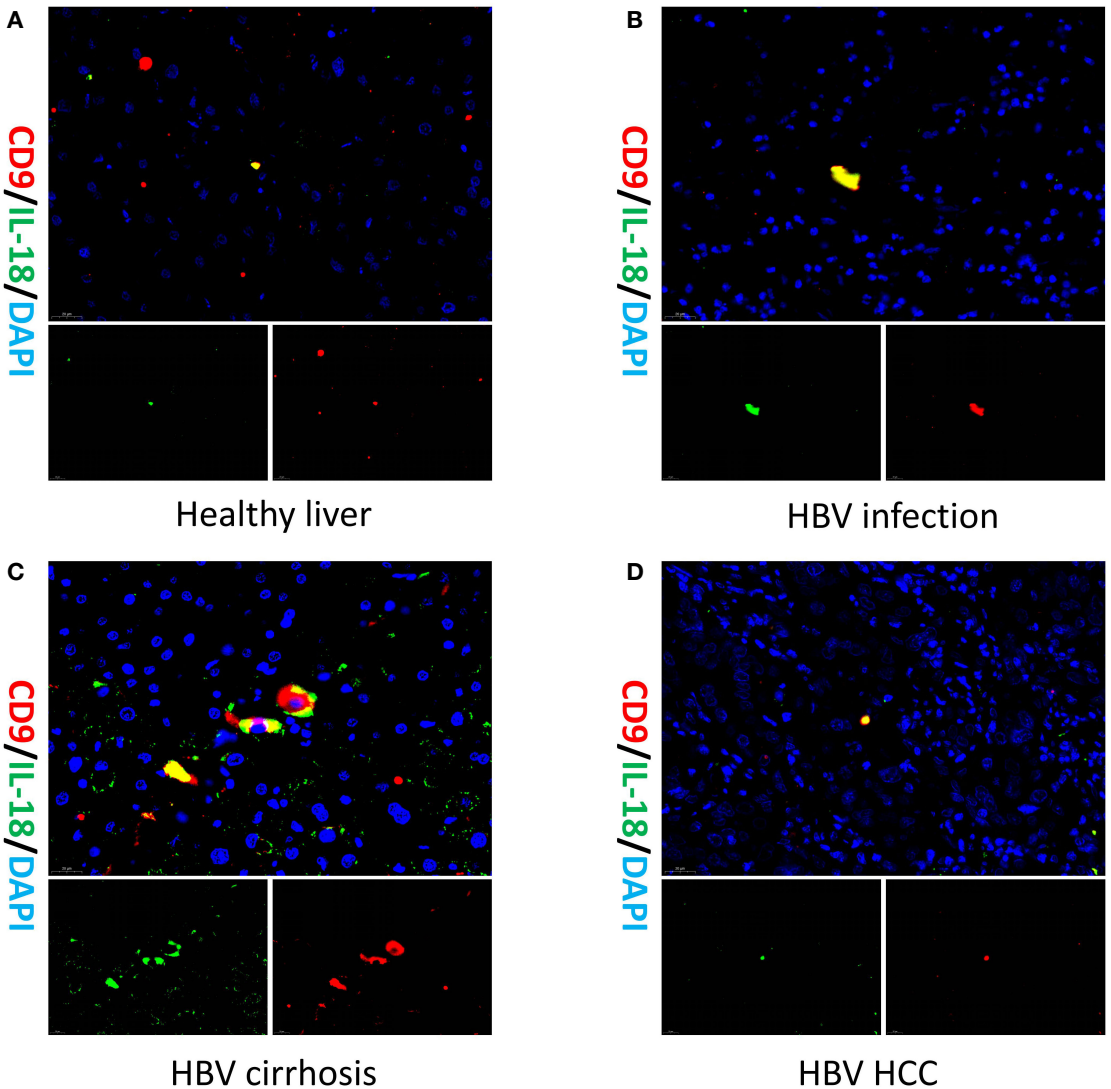
Multiplex immunofluorescence of Macrophage-CD9/IL18 in different liver diseases. Multiplex immunofluorescence images show Macrophage-CD9/IL18 in liver tissues from **(A)** healthy liver; **(B)** HBV infection; **(C)** HBV cirrhosis; **(D)** HBV HCC (hepatocellular carcinoma).

To validate the existence of the Macrophage-CD9/IL18 population and to understand their localization in healthy and diseased liver, we performed multiplex immunofluorescence of liver tissue from healthy liver, HBV infection, HBV cirrhosis, and HBV HCC (**Figure 4**). The result of multiplex immunofluorescence showed that Macrophage-CD9/IL18 exists in the above healthy and liver disease tissues (**Figure 4**).

### MAPK, ERBB, and P53 signaling pathways were gradually inhibited in myeloid cells

We conducted a GSEA analysis on myeloid cells in different disease stages (**Supplementary Figures 1A–F**). In comparison to healthy liver, HBV infection was characterized by the activation of ribosome and oxidative phosphorylation, while the suppression of p53 signaling, toll-like receptor signaling, ERBB signaling, RIG-I-like receptor signaling, and MAPK signaling was observed (**Supplementary Figures 1G,H**). In comparison between HBV infection and HBV cirrhosis, cell activation involved in immune response, exocytosis, tertiary granule, myeloid leukocyte-mediated immunity, and actin cytoskeleton were inhibited (**Supplementary Figures 1I, J**). In HBV HCC, lysosome and antigen processing and presentation were activated, while ERBB signaling, p53 signaling, MAPK signaling, and ribosome were inhibited (**Supplementary Figures 1K, L**).

### NK Cell-GNLY expanded from cirrhosis to HCC

**Figure 5:**
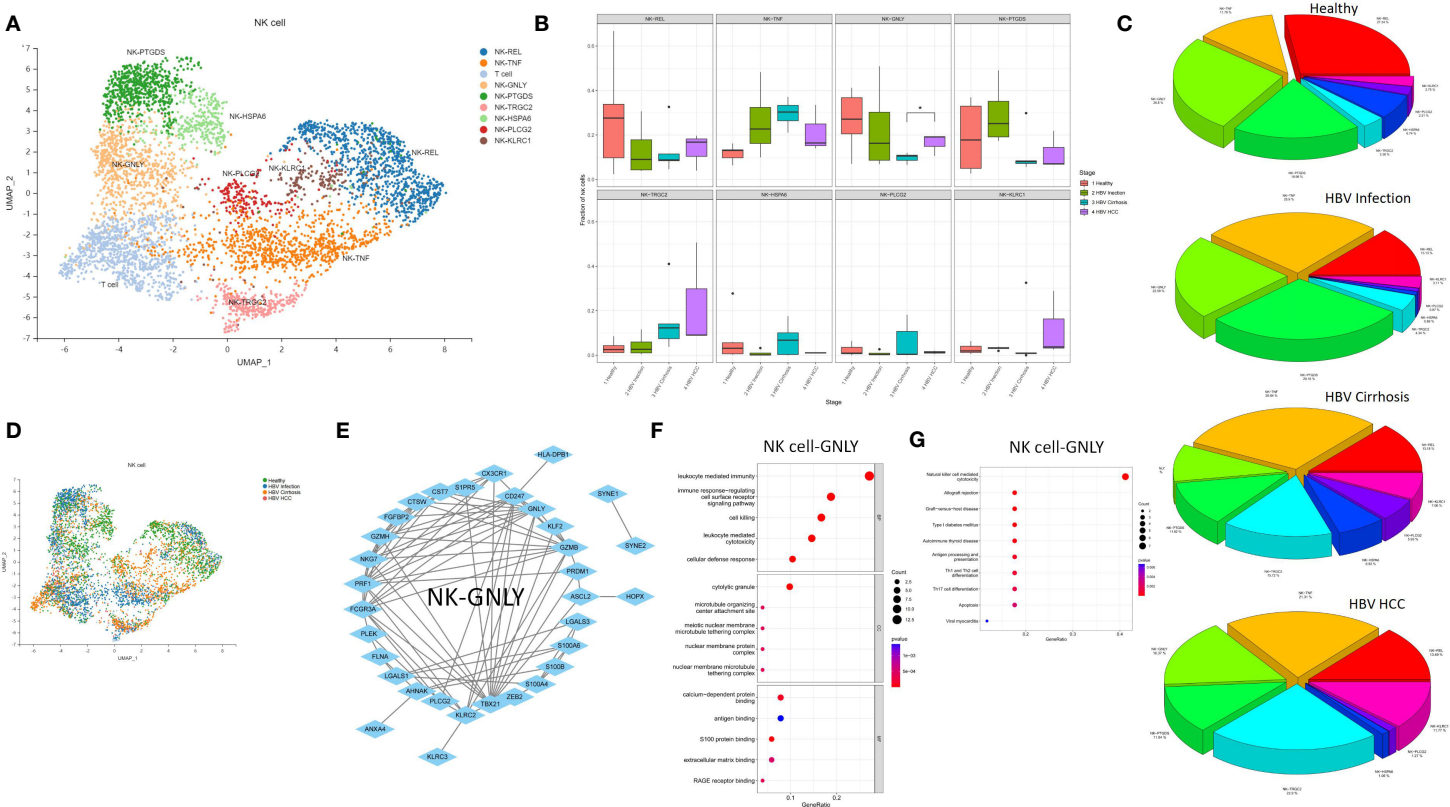
The subtypes of NK cells. **(A)** Clustering analysis of all the NK cells. **(B)** The proportion of NK cells from healthy liver to HBV infection, HBV cirrhosis, and HBV HCC located in different clusters. *p<0.05. **(C)** The average proportion of NK cells located from healthy liver to HBV infection, HBV cirrhosis, and HBV HCC. **(D)** Clustering analysis of the NK cells from healthy liver to HBV infection, HBV cirrhosis, and HBV HCC. **(E)** PPI network of the marker genes of NK cell-GNLY. **(F)** GO enrichment analysis of NK cell-GNLY marker genes. **(G)** KEGG enrichment analysis of NK cell-GNLY marker genes.

The expansion of NK Cell-GNLY from cirrhosis to HCC suggests a significant involvement of the NK cell population in the progression of cirrhosis. Eight NK cell subclusters, including NK Cell-REL, NK Cell-TNF, NK Cell-GNLY, NK Cell-PTGDS, NK Cell-TRGC2, NK Cell-HSPA6, NK Cell-PLCG2, and NK Cell-KLRC1, were identified (**Figure 5A**). The proportion of NK Cell-GNLY was found to increase in HBV HCC compared to HBV cirrhosis (P<0.05) (**Figures 5B–D**). We constructed a PPI network of the marker genes of NK Cell-GNLY (**Figure 5E**). GNLY protein, a member of the saposin-like protein family, exists in the cytotoxic particles of cytotoxic T lymphocytes and natural killer cells, which are critical for HBV clearance (14, 15). NK Cell-GNLY appears to represent a highly cytotoxic immune effector cell subset. The KEGG pathway and GO enrichment analysis of the marker genes of NK Cell-GNLY indicated that the function of NK Cell-GNLY is closely related to immune response, leukocyte activation, and cytotoxicity (**Figures 5F, G**).

### Effector CD8+ T cells expanded from HBV infection to cirrhosis and exhausted CD8+ T cell expanded from cirrhosis to HCC

**Figure 6:**
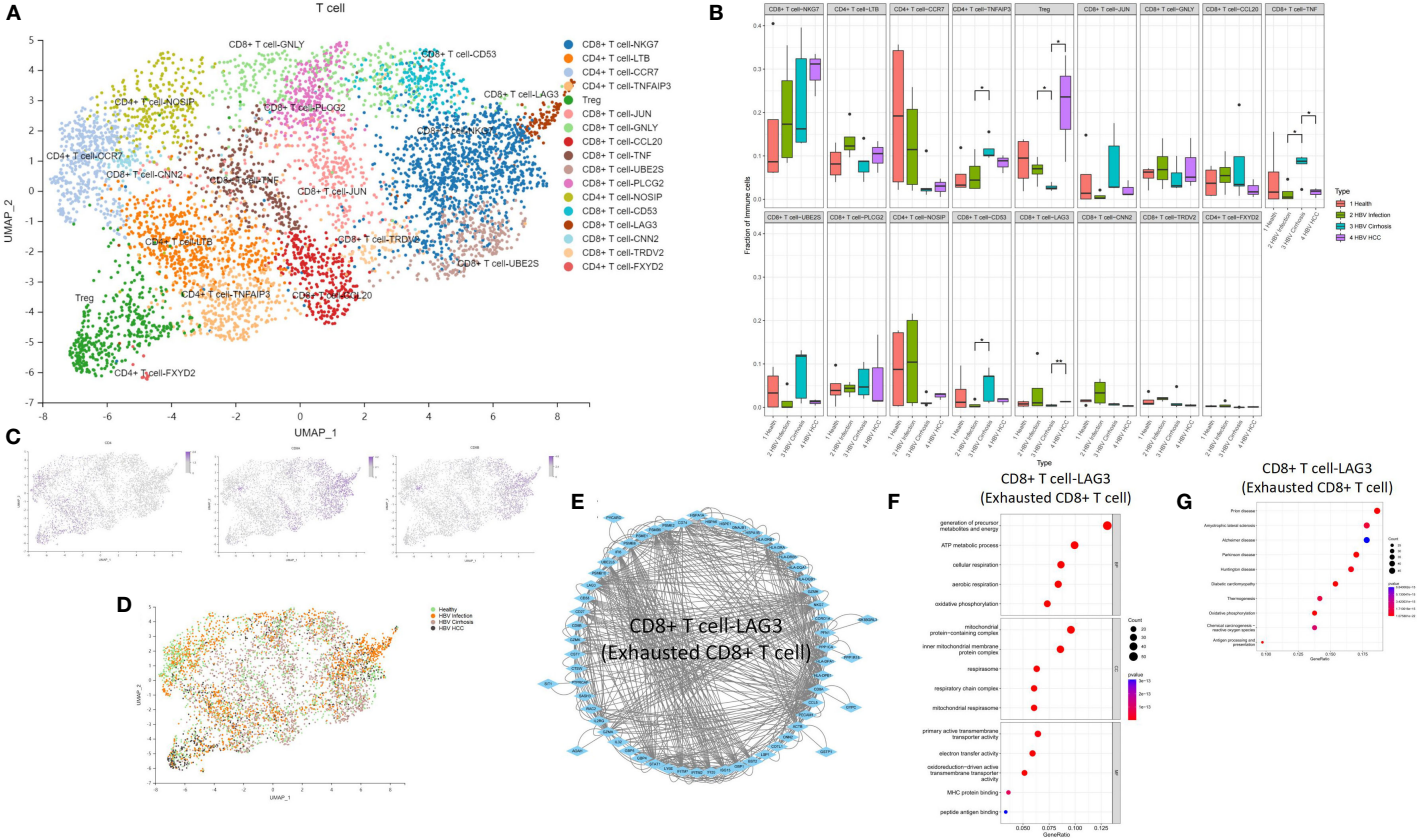
The subtypes of T cells. **(A)** Clustering analysis of all the T cells. **(B)** The proportion of T cells from healthy liver to HBV infection, HBV cirrhosis, and HBV HCC located in different clusters. *p<0.05, **p<0.01. **(C)** The expression of CD4, CD8A, and CD8B in UMAP plot. **(D)** Clustering analysis of the T cells from healthy liver to HBV infection, HBV cirrhosis, and HBV HCC. **(E)** PPI network of the marker genes of CD8 T cell-LAG3 (Exhausted CD8 T cell). **(F)** GO enrichment analysis of CD8 T cell-LAG3 (Exhausted CD8 T cell) marker genes. **(G)** KEGG enrichment analysis of CD8 T cell-LAG3 (Exhausted CD8 T cell) marker genes.

We performed an analysis of the T cell population and identified 17 T cell subclusters, including CD8 T cell-NKG7, CD4 T cell-LTB, CD4 T cell-CCR7, CD4 T cell-TNFAIP3, Treg cell, CD8 T cell-JUN, CD8 T cell-GNLY, CD8 T cell-CCL20, CD8 T cell-TNF, CD8 T cell-UBE2S, CD8 T cell-PLCG2, CD4 T cell-NOSIP, CD8 T cell-CD53, CD8 T cell-LAG3, CD8 T cell-CNN2, CD8 T cell-TRDV2, and CD4 T cell-FXYD2 (**Figure 6A**). We found that the proportion of CD4 T cell-TNFAIP3, CD8 T cell-TNF, and CD8 T cell-CD53 increased, and the proportion of Treg cells decreased in comparison between HBV infection and HBV cirrhosis (**Figures 6B–D**). TNFAIP3, also known as A20, is a potent regulator of ubiquitin-dependent signals (16). CD4 T cell-TNFAIP3 has TNFAIP3 as a marker gene. TNFAIP3 is known to promote the survival of CD4 T cells (17) and to ameliorate the severity of nonalcoholic steatohepatitis (18). CD8 T cell-TNF has TNF as a marker gene. CD8 T cell-TNF was identified as the likely effector CD8 T cell population, promoting the killing of HBV-infected cells. The proportion of effector CD8 T cells (CD8 T cell-TNF) in T cells increases in HBV cirrhosis in comparison to HBV infection. CD8 T cell-CD53 has CD53 as a marker gene. CD53 is a member of the tetraspanin superfamily that can form tetraspanin-enriched microdomains that regulate various cellular processes such as adhesion, migration, signaling, and cell fusion, and it promotes lymphocyte recirculation (19). We identified Treg cells through the marker gene FOXP3. Treg cells are a subset of immune cells specialized in suppressing excessive immune activation and maintaining immune homeostasis (20). Treg cells play an anti-fibrotic role in chronic HCV and HIV-1 infection in the liver (21, 22). We observed a decrease in their proportions between HBV infection and HBV cirrhosis livers, suggesting that the ability of Treg cells to suppress excessive immune activation and anti-fibrosis gradually weakened from HBV infection to HBV cirrhosis. The decrease of Treg cells could possibly also explain the expansion of effector T cells in cirrhosis. We have also observed an expansion of Treg cells in the progression from HBV cirrhosis to HBV HCC. In humans, a large number of tumor-infiltrating Treg cells have been found in tumors of the head and neck, breast, lung, liver, gastrointestinal tract, pancreas, and ovaries (23). Treg cells are typically associated with tumor progression and reduced survival rates in cancer patients (24).

The proportion of Treg cells and CD8 T cell-LAG3 (exhausted CD8 T cells) increased, while CD8 T cell-TNF decreased during the development of HBV HCC (**Figures 6B–D**). CD8 T cell-LAG3 was identified as exhausted CD8 T cells due to their characteristic high expression of the inhibitory immune checkpoint gene LAG3 (25). An exhausted T-cell phenotype can occur when antigen clearance fails, and exposure is maintained, such as chronic infection or cancer. A significant feature of exhausted T cells is the persistent co-expression of multiple inhibitory surface receptors, commonly referred to as immune checkpoints (26). We observed the conversion of effector CD8 T cells to exhausted CD8 T cells. For example, the proportion of effector CD8 T cells decreased, and the proportion of exhausted CD8 T cells increased in HBV HCC compared to HBV cirrhosis. Additionally, we also found that CD8+ T-NKG7 also express PD1 as a marker gene. However, the proportion of CD8+ T-NKG7 remains unchanged throughout the progression from HBV infection to cirrhosis and HCC. A PPI network of the marker genes of CD8 T cell-LAG3 was created (**Figure 6E**), and the KEGG pathway and GO enrichment analysis indicated that the function of CD8 T cell-LAG3 is closely related to oxidative phosphorylation and antigen processing and presentation (**Figures 6F, G**).

## Discussion

**Figure 7:**
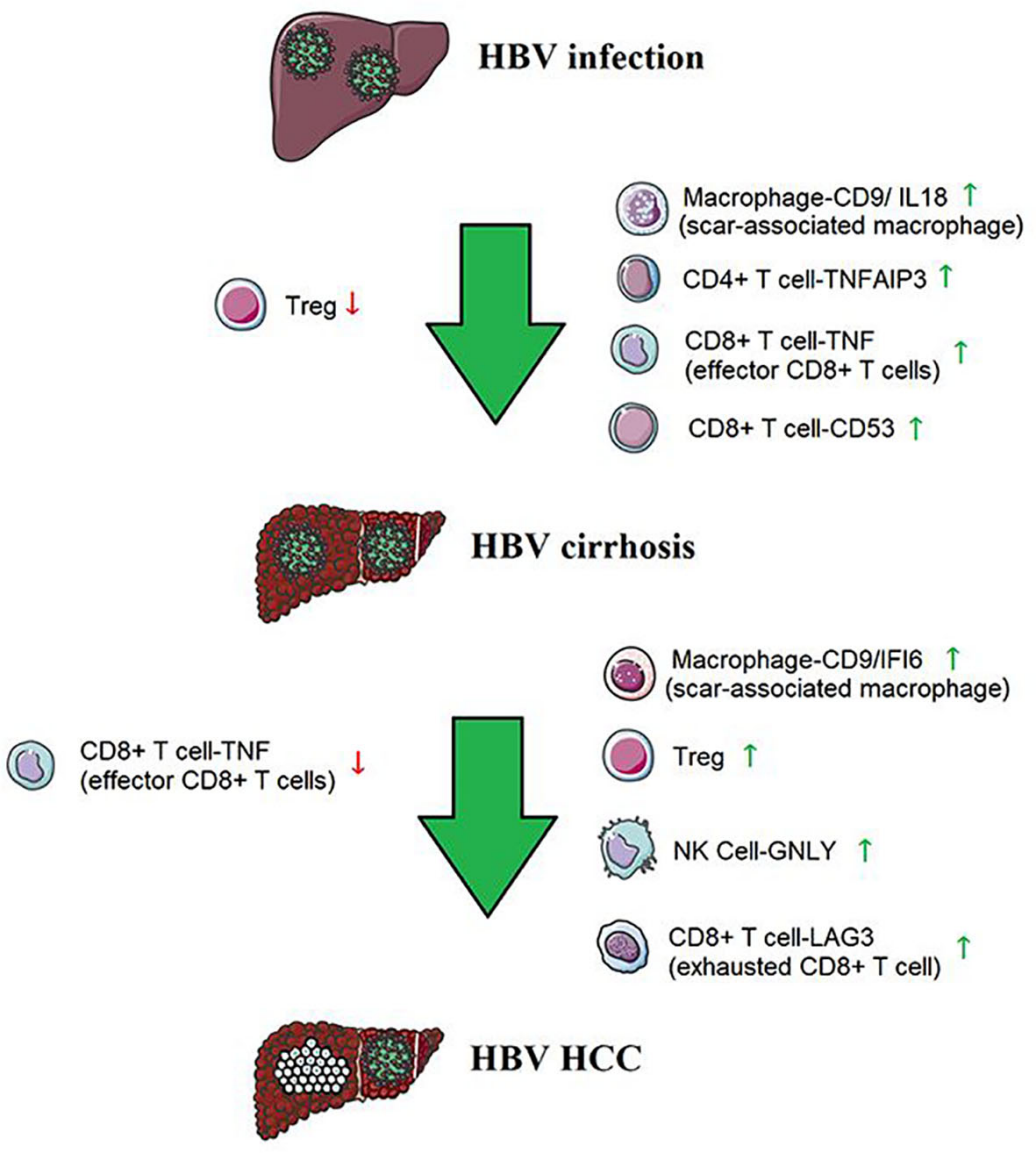
The immune cells from HBV infection to HBV cirrhosis and HBV HCC.

Our single-cell analysis revealed a unique immune ecosystem in patients’ livers from HBV infection to HBV cirrhosis and HBV HCC. Our results reveal an accumulation of Macrophage-CD9/IL18, CD4 T cell-TNFAIP3, CD8 T cell-TNF (effector CD8 T cells), and CD8 T cell-CD53 and a loss of Treg cells from HBV infection to cirrhosis, as well as an accumulation of Macrophage-CD9/IFI6, NK Cell-GNLY, Treg cell, and CD8 T cell-LAG3 (exhausted CD8 T cell) and a loss of CD8 T cell-TNF (effector CD8 T cells) from HBV cirrhosis to HCC (**Figure 7**). These results potentially indicate that changes in immune cell subsets determine, at least in part, the course from HBV infection to HBV cirrhosis and HBV HCC. And it has provided a good supplement and validation to our previous research on cirrhosis (27). Our study is an essential step in understanding how the immune environment affects HBV infection, HBV cirrhosis, and HBV HCC and reveals changes in liver immune cells from HBV infection to HBV cirrhosis and HBV HCC.

Myeloid cells are believed to be central to the pathogenesis of chronic liver injury (28), and we confirmed their close association with the disease course. The proportion of a subset of scar-associated macrophages (Macrophage-CD9/IL18) increased from HBV infection to HBV cirrhosis. Macrophage-CD9/IL18 enhanced cell communication with other immune cells from HBV infection to HBV cirrhosis. We also found another subset of scar-associated macrophages (Macrophage-CD9/IFI6) expanded from HBV cirrhosis to HBV HCC, suggesting a promoting role for Macrophage-CD9/IFI6 from HBV cirrhosis to HBV HCC. The marker genes of scar-associated macrophages in non-hepatitis cirrhosis were TREM2 and CD9 (8). However, we only find scar-associated macrophages with CD9 as a marker gene in HBV-related diseases, HBV infection, HBV cirrhosis, and HBV HCC, which may be due to the changes in the characteristics of scar-associated macrophages caused by HBV infection.

NK cells represent a subset of T lymphocytes with high abundance in the healthy liver. Classical NK cells elicit cytotoxic effects, especially in virus-infected cells, and the elimination of HBV-infected hepatocytes is driven by NK cells (29). Granulysin (GNLY) is found in cytotoxic granules of cytolytic T and NK cells (15). We found highly cytotoxic NK cells (NK Cell-GNLY) expanded from HBV cirrhosis to HBV HCC, which may be due to tumor stimulation of liver immunity.

HBV-specific effector CD8 T cells play an important role in the clearance of acute HBV infection and HBV-related liver injury (30). HBV-specific effector CD8 T cells produce anti-HBV viral cytokines and kill HBV-infected hepatocytes. We found CD8 T cell-TNF (effector CD8 T cells), and CD8 T cell-CD53 expand from HBV infection to HBV cirrhosis. T cell exhaustion is a state of T cell dysfunction that occurs during many chronic infections and cancers (31); during this process CD8 T cell-TNF (effector CD8 T cells) gradually transform into CD8 T cell-LAG3 (exhausted CD8 T cell) from HBV cirrhosis to HBV HCC. We re-characterized the function and marker genes of exhausted CD8 T cell in HBV infection, HBV cirrhosis, and HBV HCC for further studies. CD8 T cell-CD53 expanded from HBV infection to HBV cirrhosis like CD8 T cell-TNF (effector CD8 T cells), and the proportion did not change from HBV cirrhosis to HBV HCC. The function of CD8 T cell-CD53 may only be related to HBV cirrhosis but not to HBV HCC. CD4 T cell-TNFAIP3 also expanded from HBV infection to HBV cirrhosis. TNFAIP3 could promote the survival of CD4 T cells (17), and the expansion of CD4 T cell-TNFAIP3 cells promotes immune cell survival and copes with liver injury in HBV cirrhosis.

Treg cells specialize in suppressing excessive immune activation (20) by secretion of immunosuppressive cytokines and killing of effector cells or dendritic cells (25). In our research, we observed Treg cell loss from HBV infection to HBV cirrhosis, which might lead to the expansion of CD8 T cells. However, from HBV cirrhosis to HBV HCC, Treg cell expanded, leading to the transformation of CD8 T cell-TNF (effector CD8 T cells) into CD8 T cell-LAG3 (exhausted CD8 T cell).

In conclusion, we comprehensively compared scRNA-seq data of immune cells between healthy livers, HBV infection, HBV cirrhosis, and HBV HCC. The results depicted the unique immune ecosystem from HBV infection to HBV cirrhosis and HBV HCC, and may justify future studies for a deeper understanding of the mechanisms driving the development of disease progression from healthy liver to HBV cirrhosis and HCC.

## Data availability statement

The datasets presented in this study can be found in online repositories. The names of the repository/repositories and accession number(s) can be found below: https://www.ncbi.nlm.nih.gov/, PRJNA833766.

## Ethics statement

The studies were conducted in accordance with the local legislation and institutional requirements. The study was approved by the First Affiliated Hospital of Harbin Medical University Ethics Review Center (approval number. 2021179). The participants provided their written informed consent to participate in this study.

## Author contributions

QB: Conceptualization, Data curation, Formal analysis, Funding acquisition, Investigation, Methodology, Project administration, Resources, Software, Supervision, Validation, Visualization, Writing – original draft, Writing – review & editing. RL: Data curation, Writing – original draft. XHe: Data curation, Writing – original draft. XHo: Writing – original draft. YY: Writing – original draft. ZZ: Writing – original draft. HL: Writing – original draft. FT: Writing – original draft. CE: Writing – original draft. TH: Writing – original draft.

## Supplementary material

Supplementary Figure 1 GSEA analysis of myeloid cells. **(A)** GSEA GO analysis of myeloid cells from health to HBV infection. **(B)** Five inhibition and five promotion result in myeloid cell GSEA GO analysis from health to HBV infection. **(C)** GSEA GO analysis of myeloid cells from HBV infection to HBV cirrhosis. **(D)** Five inhibition and five promotion result in myeloid cell GSEA GO analysis from HBV infection to HBV cirrhosis. **(E)** GSEA GO analysis of myeloid cells from HBV cirrhosis to HBV HCC. **(F)** Five inhibition and five promotion result in myeloid cell GSEA GO analysis from HBV cirrhosis to HBV HCC. **(G)** GSEA KEGG analysis of myeloid cells from health to HBV infection. **(H)** Five inhibition and five promotion result in myeloid cell GSEA KEGG analysis from health to HBV infection. **(I)** GSEA KEGG analysis of myeloid cells from HBV infection to HBV cirrhosis. **(J)** Five inhibition and five promotion result in myeloid cell GSEA KEGG analysis from HBV infection to HBV cirrhosis. **(K)** GSEA KEGG analysis of myeloid cells from HBV cirrhosis to HBV HCC. **(L)** Four inhibition and five promotion result in myeloid cell GSEA KEGG analysis from HBV cirrhosis to HBV HCC.
