# Supplementary figures and images for: Single-cell landscape of immune cells during the progression from HBV infection to HBV cirrhosis and HBV-associated hepatocellular carcinoma
Source: Front Immunol. 2023 Dec 5;14:1320414. doi: 10.3389/fimmu.2023.1320414 (PMC10729758; doi:10.3389/fimmu.2023.1320414)

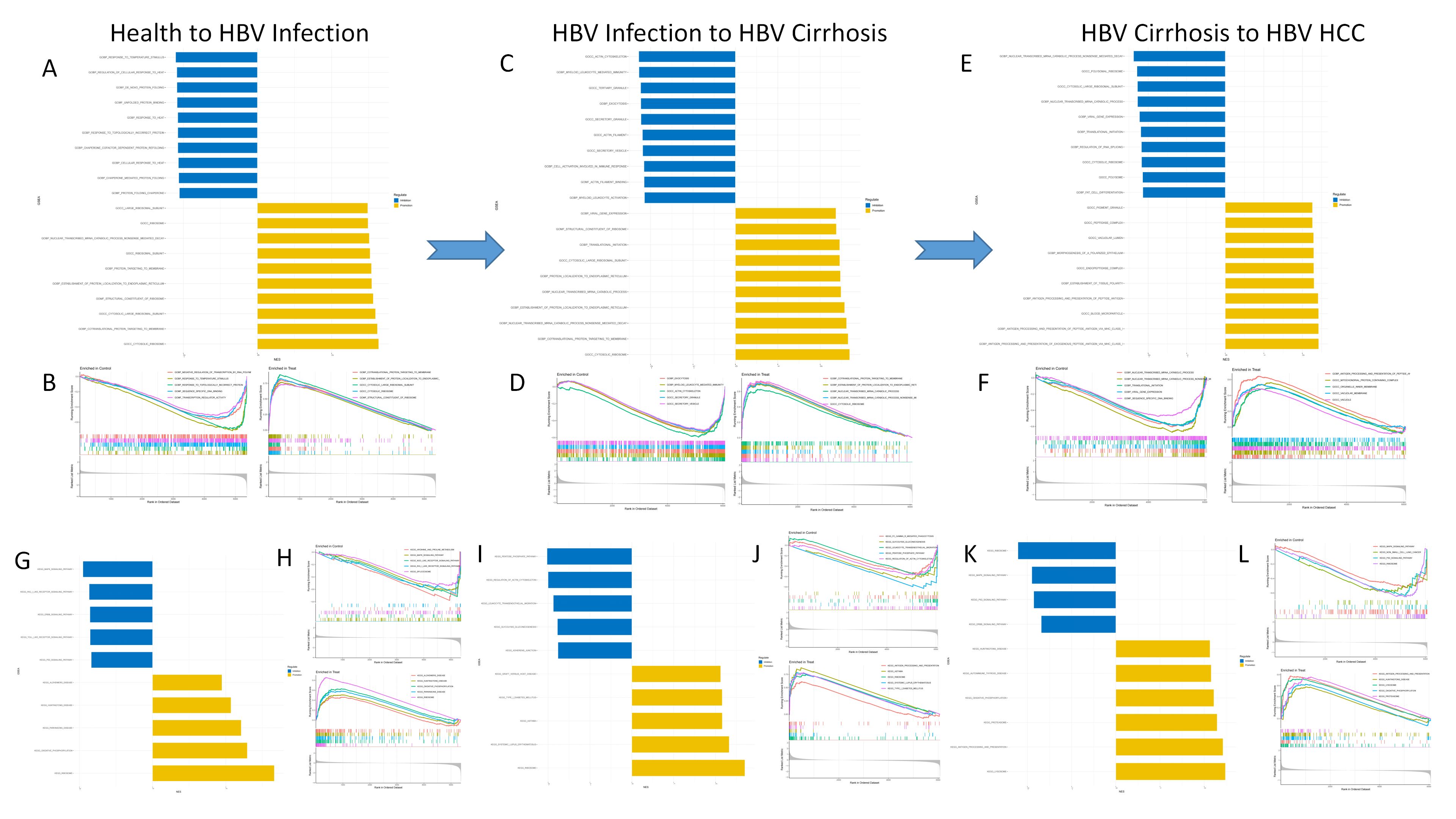

Supplement: Supplementary Figure 1 — GSEA analysis of myeloid cells. (A) GSEA GO analysis of myeloid cells from health to HBV infection. (B) Five inhibition and five promotion result in myeloid cell GSEA GO analysis from health to HBV infection. (C) GSEA GO analysis of myeloid cells from HBV infection to HBV cirrhosis. (D) Five inhibition and five promotion result in myeloid cell GSEA GO analysis from HBV infection to HBV cirrhosis. (E) GSEA GO analysis of myeloid cells from HBV cirrhosis to HBV HCC. (F) Five inhibition and five promotion result in myeloid cell GSEA GO analysis from HBV cirrhosis to HBV HCC. (G) GSEA KEGG analysis of myeloid cells from health to HBV infection. (H) Five inhibition and five promotion result in myeloid cell GSEA KEGG analysis from health to HBV infection. (I) GSEA KEGG analysis of myeloid cells from HBV infection to HBV cirrhosis. (J) Five inhibition and five promotion result in myeloid cell GSEA KEGG analysis from HBV infection to HBV cirrhosis. (K) GSEA KEGG analysis of myeloid cells from HBV cirrhosis to HBV HCC. (L) Four inhibition and five promotion result in myeloid cell GSEA KEGG analysis from HBV cirrhosis to HBV HCC. [file Image_1.jpeg]
